# Aberrant amygdala functional connectivity at rest in pediatric anxiety disorders

**DOI:** 10.1186/s13587-014-0015-4

**Published:** 2014-12-09

**Authors:** Lisa L Hamm, Rachel H Jacobs, Meghan W Johnson, Daniel A Fitzgerald, Kate D Fitzgerald, Scott A Langenecker, Christopher S Monk, K Luan Phan

**Affiliations:** Department of Psychiatry, University of Illinois at Chicago, 1747 W. Roosevelt Road, IJR/WROB Rm. 244, Chicago, IL 60608 USA; Department of Psychiatry, University of Michigan, Ann Arbor, Michigan USA; Department of Psychology, University of Michigan, Ann Arbor, Michigan USA; Department of Psychology, University of Illinois at Chicago, Chicago, Illinois USA; Department of Anatomy and Cell Biology, University of Illinois at Chicago, Chicago, Illinois USA

**Keywords:** Children, Adolescent, Anxiety, Amygdala, Connectivity, Resting state, fMRI

## Abstract

**Background:**

Childhood onset of anxiety disorders is associated with greater functional impairment and burden across the lifespan. Recent work suggests that generalized anxiety disorder (GAD) is characterized by dysfunctional connectivity in amygdala-based circuits at rest in adolescents, consistent with adults. However, neural mechanisms underlying a broad spectrum of often-comorbid anxiety disorders in children remains unclear and understudied. The current study examines amygdala functional connectivity at rest in children and adolescents across comorbid anxiety disorders (ADs) including youth with primary diagnoses of GAD and social phobia (SP).

**Results:**

Compared with healthy controls (HCs), AD youth exhibited hyperconnectivity between the right amygdala and the insula and hypoconnectivity between the left amygdala and the ventromedial prefrontal cortex (vmPFC) and posterior cingulate cortex (PCC). Within the AD group, connectivity was not correlated with anxiety severity and only the amygdala-PCC connectivity was positively correlated with age.

**Conclusions:**

Our findings indicate that youth with comorbid ADs demonstrate aberrant connectivity in the anterior limbic network (ALN) as well as the PCC at rest. This extends upon previous work suggesting alterations in amygdala circuits underlying fear learning, emotion regulation, and the processing of interoceptive states. Presence of these findings within this young, comorbid sample points to underlying common mechanisms across ADs and illuminates future targets for prevention and intervention in childhood.

## Background

Anxiety disorders (ADs) are among the most prevalent and disabling psychiatric disorders to occur in youth [1-4] and set children on a negative trajectory towards continued and additional comorbid psychological disorders during adulthood [5-7]. When left untreated, pediatric anxiety disorders can result in severe ongoing social impairment, decreased educational achievement, and interrupted employment [7-9]. The three most common anxiety disorders among youth include generalized anxiety disorder (GAD), social phobia (SP), and separation anxiety disorder (SAD) and are collectively referred to as the “pediatric anxiety triad” [10,11]. High rates of comorbidity across these diagnoses suggest greater similarities than differences [12], including sensitivity to perceived or actual negative life events [13] and debilitating worry leading to avoidance patterns (DSM-5, 2013). Additionally, these three disorders respond to similar treatments [12,14,15], further implicating diagnostic overlap and, perhaps, common neural mechanisms. Recent examinations of pediatric anxiety have moved towards a more dimensional approach by including children with comorbid ADs to evaluate neural correlates [16], as well as the effectiveness of treatments, such as cognitive behavioral therapy (CBT), in reducing the severity of anxiety symptoms [17].

Despite the prevalence and negative sequelae of the pediatric anxiety triad, research examining the underlying neural mechanisms is in its infancy. The amygdala is the most frequently studied region of interest in pediatric anxiety, given the robust human neuroimaging literature documenting amygdala activity and connectivity as it relates to emotional processing and regulation [18,19]. Indeed, amygdala hyperactivation to perceived threat is a defining neuropathophysiological feature of anxiety disorders [20-22] and frontal regions are known to have dense bidirectional structural connections with the amygdala [23,24]. The amygdala is one region contributing to what has been labeled the anterior limbic network (ALN; [18]). This network encompasses connections between the amygdala, medial prefrontal cortex (mPFC), insula, anterior cingulate cortex (ACC), as well as the ventrolateral and dorsolateral prefrontal cortexes (vlPFC, dlPFC) [18]. These regions modulate complex social and emotional behaviors and share architectural and functional features [25]. Reciprocal connections within this network are hypothesized to contribute to monitoring of internal and external sensory information in order to maintain emotional equilibrium [26].

The strongest evidence implicating aberrant ALN function in anxiety disorders derives from several task-based fMRI studies that measure connectivity of networks during emotional tasks. Altered functional connectivity patterns have been observed during emotional processing and fear responding in regions composing the ALN among adults with anxiety [27,28] as well as among youth [19,22,29,30]. Adults with ADs have demonstrated decreased connectivity between the amygdala and the rostral ACC and dlPFC while viewing fearful faces [27]. An examination of functional connectivity during a face-emotion rating task found greater connectivity between the right amygdala and the insula in youth with GAD compared to healthy controls (HCs). Anxiety symptom severity (as measured by the Pediatric Anxiety Rating Scale (PARS)) was correlated with extent of with amygdala-insula connectivity [29]. Functional connectivity studies of both adults and youth support the notion that ALN disruption contributes to symptoms of anxiety. Disruptions in this network may underlie core phenotypic features of the disorder across the lifespan [31].

Functional connectivity can also be measured during the resting state (labeled rs-fMRI) and allows for the examination of the intrinsic functional connectivity (iFC) *in the absence of a specific emotional task*. Rs-fMRI has proven useful in interrogating neural circuits implicated in anxiety-related processes, with several studies demonstrating the existence of disrupted connectivity *at baseline* in amygdala-based networks among adults with anxiety disorders [27,32]. Importantly, iFC methods have yielded reliable individual differences in neural connectivity that are correlated with self-report of behavior and symptoms [33-36]. This technique has been utilized in recent studies of healthy adults to demonstrate several iFC patterns that covaried with positive and negative affect [35], and trait levels of anxiety modulated amygdala-mPFC connectivity [37]. These results implicate the relevance of functional connectivity in the affective domain *even in the absence of an emotional challenge* among adults with anxiety disorders. In addition, recent rs-fMRI studies have demonstrated altered resting state connectivity in regions considered part of the ALN, including the ACC, mPFC, and insula [27,38,39].

A region outside of the ALN that has been implicated in social and general anxiety is the posterior cingulate cortex (PCC) and the adjacent precuneus. The PCC in particular may play a role in emotional evaluation [40] and social behavior [41]. The PCC may also contribute to modulation of the amygdala [42]. Rs-fMRI data collected from adults with anxiety disorders found that reduced connectivity between the amygdala and the PCC/precuneus was associated with higher state anxiety [43]. Among adolescents with GAD, one study that has examined connectivity of the amygdala during a task of emotional and neutral images found altered connectivity between the right amygdala and the posterior cingulate [19]. In sum, task-based fMRI studies have identified abnormalities in the PCC among youth with anxiety disorders [19,29], but limited work has examined this region at rest among youth.

Examinations of connectivity among youth with anxiety disorders is understudied to date, partly due to the difficulty in recruiting this population and acclimatizing them to the fMRI environment. However, the altered connectivity patterns observed among adults may not be applicable to pediatric populations due to the important structural and functional developmental changes known to occur in the brain during childhood and adolescence [44-46]. Examining the developmental trajectory of neural network abnormalities among youth with anxiety may elucidate predictive or modifiable biomarkers of anxiety in addition to illustrating the long-term effects of anxiety on neurodevelopment [47]. To the best of our knowledge, only one study to date has used rs-fMRI to examine functional connectivity in youth with anxiety [48]. This study documented perturbations in connectivity between the amygdala and the following regions: ACC, striatum, insula, superior temporal gyrus, as well as prefrontal regions including the ventromedial prefrontal cortex (vmPFC), dmPFC, vlPFC, and dlPFC among fifteen youth between 12 and 17 with a diagnosis of GAD. These differences support a more widespread disruption of network function than previously identified.

In the present study, we sought to contribute to the literature by examining rs-fMRI using bilateral amygdala seeds in a sample of 33 youth with primary ADs of GAD and/or SP with several comorbidity profiles and compared them to data for 23 healthy controls (ages 7 to 19). We chose to examine both the left and right amygdala seeds separately given the only pediatric anxiety rs-fMRI study to date detected laterality in amygdala connectivity [48]. We sought to study a representative heterogeneous diagnostic group that would allow for greater generalizability of findings consistent with epidemiologic and intervention trials that demonstrate comorbidity across these disorders and commonality in treatment response [14,49]. In line with emotion regulation models of ADs [50], we hypothesized that relative to healthy peers, youth with ADs would demonstrate hyperconnectivity between the amygdala and insula. We also hypothesized that youth with AD would demonstrate hypoconnectivity between the amygdala and regions included in the ALN such as the ACC and mPFC. We also sought to explore amygdala-PCC connectivity but did not hypothesize a direction based on the paucity of findings to date.

## Methods

### Subjects

Participants included 33 children (mean age 13.9 ± 3.1 years; 22 female) with a DSM-IV (1) primary diagnosis of GAD, SP, and SAD (collectively referred to as ADs) who were compared to 23 matched HCs (14.6 ± 3.9 years; 13 female; Table 1). All participants were medication-free at the time of testing and were recruited from the University of Michigan Pediatric Anxiety Disorders Clinic as well as advertisements posted in the local community. All subjects had negative urine drug test (and pregnancy test for females) which were administered immediately prior to the fMRI scan. Exclusion criteria included an IQ below 70, a lifetime diagnosis of bipolar disorder, schizophrenia, and/or a pervasive developmental disorder. Healthy comparison youth were required to be free of lifetime diagnoses of DSM-IV Axis I and II disorders. All participants provided written informed consent/assent. The study was approved by the University of Michigan Institutional Review Board.

**Table 1:** **Sample demographic data and clinical features**

	**Anxiety disorder**	**Healthy controls**	***p value***
Total (*n*)	33	23	
Sex, female, *n* (%)	22 (67)	13 (57)	
Age, mean ± SD	13.9 ± 3.1	14.6 ± 3.9	n.s.
Race	29 C, 2 AA, 2 multi	16 C, 1 AA, 1 As, 4 multi, 1 unknown	n.s.
Primary anxiety diagnoses (*n*) (percent of total)			
Generalized anxiety disorder	23 (70%)		
Social phobia	10 (30%)		
PARS ± SD	22 ± 3.9	2 ± 2.5	*p* < 0.001

*C* Caucasian, *AA* African-American, *As* Asian, *Multi* multiracial, *PARS* Pediatric Anxiety Rating Scale, *n.s.* non-significant (*p* > 0.05).

Both groups of participants (HCs and ADs) were interviewed by clinically trained mental health professionals using the Kiddie-Schedule for Affective Disorders-Present and Lifetime Version (K-SADS-PL) [51], and diagnoses were confirmed by a board-certified psychiatrist. A second rater conducted reliability on 20% of cases to establish inter-rater reliability. Within the primary GAD group, 10 participants (30%) received a comorbid SP/SAD diagnosis. Within the primary SP group, 6 participants (40%) received a comorbid GAD/SAD diagnosis. Current anxiety symptom severity and impairment was assessed by a clinician using the PARS [52] which has demonstrated high inter-rater reliability (The Research Units On Pediatric Psychopharmacology Anxiety Study Group, 2002).

### fMRI acquisition

Functional imaging was performed with blood-oxygen-level-dependent (BOLD) sensitive whole-brain fMRI on a 3.0 Tesla GE Signa System (General Electric; Milwaukee, WI) using a four-channel GE Quadrature sending and receiving head coil. Images were acquired with 30 axial, 5-mm-thick slices using a standard T2*-sensitive gradient echo reverse spiral acquisition sequence (2 s repetition time; 25 ms echo time; 64 × 64 matrix; 24 cm field of view; flip angle 77°; 3.75 × 3.75 × 5 mm final voxel size). A high-resolution, T1-weighted volumetric anatomical scan was also acquired in the axial plane (9 ms repetition time, 1.8 ms echo time; 256 × 256 matrix; 256 mm field of view; flip angle 15°; 124 slices; 1.2 mm slice thickness) at the same position as the functional images for anatomical localization and spatial normalization. Resting-state functional imaging included one 8-min scan during which participants were instructed to look at a fixation cross and let their mind wander without falling asleep.

### fMRI analysis

Images were preprocessed and analyzed with the CONN: functional connectivity toolbox (http://www.nitrc.org/projects/conn), with preprocessing steps implemented in SPM8 (http://www.fil.ion.ucl.ac.uk/spm/) running on the MATLAB (Math Works, Natick, MA) platform. Images were segmented into gray matter, white matter, and cerebrospinal fluid (CSF) for use during removal of temporal confounds. Data were then motion corrected, coregistered with a high resolution T1 image, normalized to the Montreal Neurological Institute (MNI) space, and smoothed with an 8-mm Gaussian kernel of full width at half-maximum. Importantly, patients and healthy controls did not significantly differ in either total (*p* = 0.255), maximum (*p* = 0.443) or average (*p* = 0.170) head motion. Connectivity preprocessing followed the CompCor method [53] for removal of non-neuronal sources of noise, as opposed to relying on global signal regression, which subsequently allows for interpretation of anticorrelations. Amygdala connectivity maps were generated for each subject using a seed-driven approach in which the left and right amygdala were defined by the Automated Anatomical Labeling (AAL) atlas based on the Talairach Daemon database [54]. The entire BOLD time course was extracted from the amygdala seeds, and Pearson’s correlation coefficients were calculated between these entire time courses and the time courses of all other voxels across the brain and a mean time course across the entire region of interest. Fisher transformation was used to convert the resulting correlation coefficients into *Z*-scores which were then used in second-level general linear model analyses. Group differences in amygdala connectivity were examined using two-tailed independent samples *t* tests. To determine significance within *a priori* regions in which we had a strong hypothesis within both the ALN (mPFC, ACC, and insula) and PCC given the extant literature, we restricted our analyses to these relevant regions using an anatomically derived (AAL atlas) partial brain mask of the mPFC, ACC, PCC, and insula (search volume = 131,272 mm^3^) inclusive of these four anatomical areas. For each between-group analysis in the right and left amygdala connectivity, cluster-based significance thresholding was used to adjust for multiple comparisons within the search volume. Cluster-based significance thresholding was determined via simulation using the ClusterSim utility (10,000 iterations; http://afni.nimh.nih.gov/pub/dist/doc/program_help/3dClustSim.html). Given smoothness estimates of the data (11.2 mm × 11.1 mm × 10.0 mm) across our region of interest mask with a volume of 131.3 cm^3^, a family-wise error correction at *α* < 0.05 is realized with a voxel threshold of *p* < 0.001 with minimum cluster size of 44 voxels (352 mm^3^).

### Statistics

For *a priori* areas showing group differences, we extracted parameter estimates/beta weights (connectivity values, arbitrary units) from a 10-mm spherical region of interest surrounding peak from each subject to illustrate the direction of effects. To investigate the relationship between connectivity differences and clinical symptom severity, PARS scores were correlated with connectivity values extracted from regions in which significant group differences were observed. A similar correlational analysis was conducted with age. To ensure findings did not differ by primary diagnoses, we used independent samples *t* tests to examine whether significant findings differed based on a primary diagnosis of GAD versus a primary diagnosis of SP.

## Results and discussion

### Group differences in right amygdala connectivity

**Figure 1:**
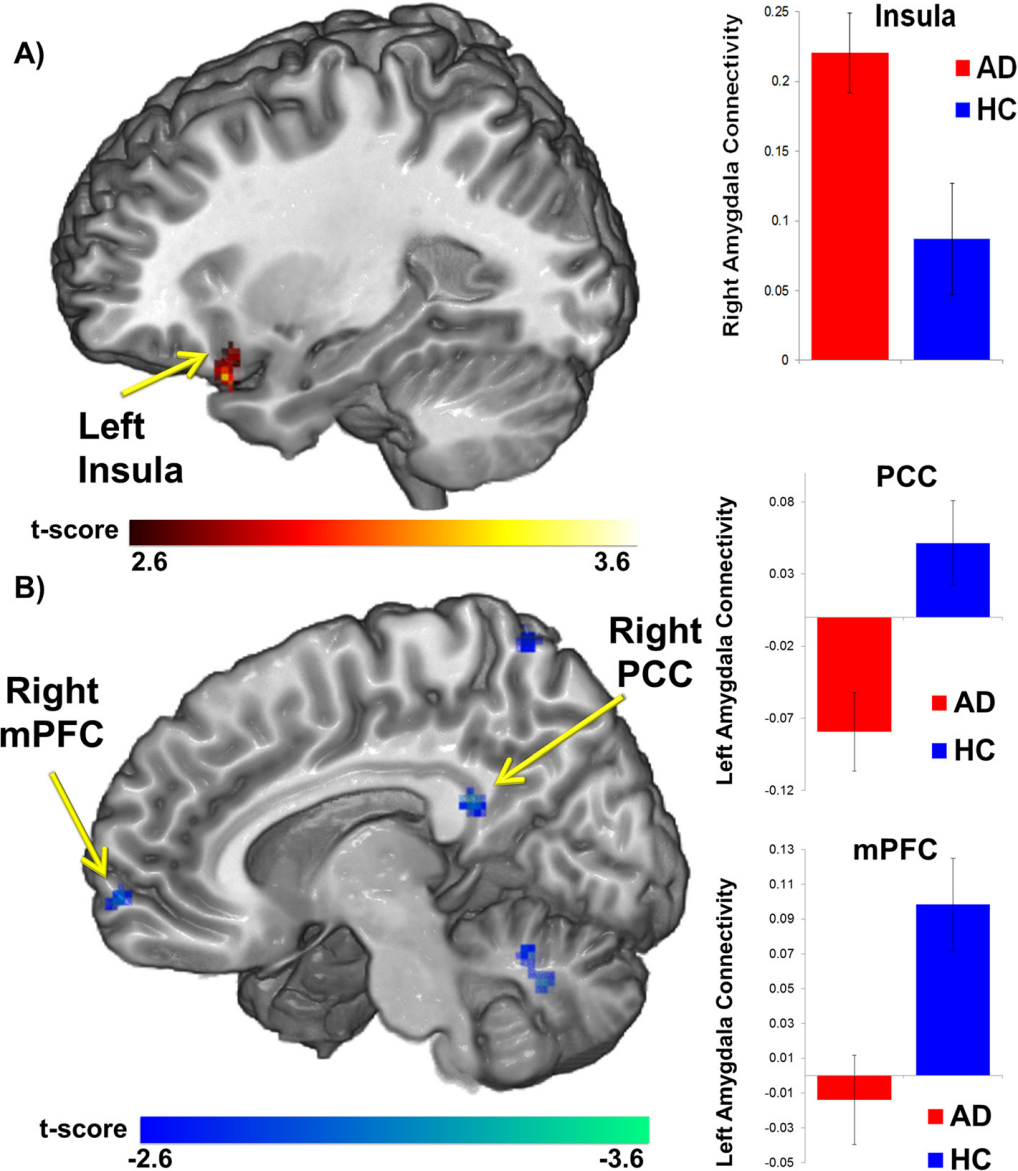
**Between-group whole-brain voxel-wise statistical** ***t*** **map of** ***a priori*** **hypothesized group differences overlaid on a canonical brain rendering showing: (A) greater right amygdala connectivity to the insula (anxiety disorder (AD) group > healthy control (HC) group; and (B) decreased amygdala connectivity to the medial prefrontal cortex (mPFC) and posterior cingulate cortex (PCC) (AD < HC).** Statistical *t* map thresholded at whole-brain voxel-wise (*p* < 0.005, cluster volume > 352 mm^3^, representing corrected *α* < 0.05); color bar represents statistical *t* scores. Bar graph shows mean extracted parameter estimate *β* weights in arbitrary units (±SEM) within each group from 10 mm spherical region of interest surrounding peak.

The AD group exhibited hyperconnectivity from the right amygdala to the left insula (BA 47, *Z*-score = 3.34, *p* < 0.05, corrected), compared to the HC group (Table 2; Figure 1A). In contrast, the AD group did not exhibit hypoconnectivity between the right amygdala to any *a priori* regions of interest, compared with the HC group. Table 2 also displays group differences in areas outside of *a priori* regions for completeness, to obviate bias and to promote new hypotheses for future studies.

**Table 2:** **Group differences in right amygdala functional connectivity at rest**

	**Region**	**MNI coordinates**			**Volume**	***T*** **value**
		***x***	***y***	***z***	**(mm** ^**3**^ **)**	
AD > HC						
	Pallidum	−12	6	0	536	3.67
	Superior temporal cortex	−54	16	−14	536	3.62
	Rectus	−4	12	−22	624	3.55
	*Insula*	−28	20	−24	688	3.34
	Brainstem/pons	−6	−10	−24	176	3.32
HC > AD						
	Cerebellum	28	−62	−40	1,512	4.10
	Fusiform cortex	−38	−48	−18	280	3.13

All listed clusters are significant at *p* < 0.005 (uncorrected) with a cluster extent threshold of greater than 20 contiguous voxels.

Areas showing *a priori* hypothesized group differences italicized.

*MNI* Montreal Neurological Institute, *AD* anxiety disorder, *HC* healthy control.

### Group differences in left amygdala connectivity

The AD group exhibited hypoconnectivity between the left amygdala and the mPFC (BA 10/11, *Z*-score = 3.18, *p* < 0.05, corrected) and the PCC (BA 26, *Z*-score = 3.69, *p* < 0.05, corrected), as compared with the HC group (Table 3; Figure 1B). In contrast, the AD group did not exhibit hyperconnectivity between the left amygdala to any *a priori* regions of interest, compared with the HC group (Table 3). Table 3 also displays group differences in areas outside of *a priori* regions for completeness, to obviate bias, and to promote new hypotheses for future studies.

**Table 3:** **Group differences in left amygdala functional connectivity at rest**

	**Region**	**MNI coordinates**			**Volume**	***T*** **value**
		***x***	***y***	***z***	**(mm** ^**3**^ **)**	
*AD > HC*						
	Cerebellum	6	−64	−46	1,128	4.02
	Brainstem–midbrain	−4	−32	−2	272	3.40
	Precentral cortex	−62	−12	40	192	3.38
	Cuneus	−8	−96	14	440	3.32
	Thalamus	−18	−24	8	168	3.14
	Ventro-lateral prefrontal cortex	56	38	10	192	3.06
*HC > AD*						
	*Posterior cingulate cortex*	10	−40	24	456	3.69
	Superior parietal cortex	16	−58	72	832	3.38
	*Medial prefrontal cortex*	6	62	−4	376	3.18
	Inferior parietal cortex	−36	−80	44	1,424	3.15

All listed clusters significant at *p* < 0.005 (uncorrected) with a cluster extent threshold of greater than 20 contiguous voxels.

Areas showing *a priori* hypothesized group differences italicized.

*MNI* Montreal Neurological Institute, *AD* anxiety disorder, *HC* healthy control.

### Amygdala connectivity and correlations with symptom severity and age

No significant correlations were observed between seed-cluster connectivity values from the insula, vmPFC, and PCC with anxiety severity among youth with ADs (all *p*s > 0.5). However, when looking across the entire sample, PARS scores were significantly negatively correlated with amygdala-PCC (*r =* −0.37, *p* < 0.01) and amygdala-vmPFC (*r* = −0.37, *p* < 0.01) connectivity values and positively correlated with amygdala-insula (*r* = 0.36, *p* < 0.01) connectivity.

In terms of age, the correlation between left amygdala-PCC connectivity and age was significant (*r* = 0.48; *p* < 0.01) among youth with AD; the correlation was not significant within the healthy control group. Increasing age was associated with increased connectivity between the amygdala and the PCC, whereas among younger children decreased connectivity between these regions was observed. Of note, even when controlling for age, the previously identified connectivity differences between groups remained.

### Comorbidity profiles

Our analysis included 10 participants (30% of the AD group) with a primary diagnosis of SP compared to 70% with a primary diagnosis of GAD; as such, we examined for group differences in connectivity between GAD and SP. The youth with a primary diagnosis of GAD did not differ from those with a primary diagnosis of SP for connectivity between the amygdala and the insula (*t*(31) = −0.05, *p* = 0.96), left PCC (*t*(31) = −0.74, *p* = 0.47), or the left vmPFC (*t*(31) = 1.47, *p* = 0.15).

## Discussion

Consistent with our hypotheses, the youth with AD demonstrated aberrant amygdala connectivity with regions of the ALN including the vmPFC and insula when compared to HCs. Surprisingly, we did not find connectivity differences with the ACC but did observe amygdala-PCC hypoconnectivity among AD compared to HC youth. Our results replicate previous observations and extend upon the only study to date that has examined resting-state iFC in adolescents with AD [48], suggesting these findings may be reliable and could even generalize across diagnostic categories - from GAD to SP and their comorbidities.

Specifically, we found hyperconnectivity between the right amygdala seed and the left insula among anxious youth compared to HC peers, consistent with the previous literature [34,55-57]. Insula and amygdala involvement in the detection of salience, emotion, and attention is well established [34] and task-based fMRI findings have indicated the hyperactivity of these regions may be a key neural mechanism underlying anxiety-related processes [34,58,59]. The amygdala has been found to be structurally connected to the insula [60], and our results contribute to emerging evidence of a functional connection between the structures [27,34,48]. Altered functional connectivity between the amygdala and insula has been previously observed in groups with anxiety disorders during task [27,29] and more recently during rest [27,38,39,48]. Given the insula’s role in interoceptive processing, increased connectivity with the amygdala at rest may reflect increased interactions between a region implicated in fear perception-expression (amygdala) and another implicated in anxious arousal-anticipation (insula).

The extant literature implicates dysfunction in amygdala connections to the prefrontal cortex [19]. Our finding of decreased iFC between the amygdala and frontal regions such as the vmPFC among youth with ADs is consistent with prior findings in adult and pediatric resting-state studies. Specifically, previous research in healthy adults has demonstrated positive coupling between the amygdala and vmPFC at rest [37,61] and has also found this relationship to be compromised in those with higher levels of self-reported anxiety [37]. The latter study found those with high levels of anxiety displayed negative coupling between the amygdala and vmPFC. These findings have since been replicated within a sample of adolescents with GAD [48]. This study documented perturbed amygdala-PFC circuitry, finding negative connectivity between amygdala and vmPFC and positive connectivity between amygdala and dmPFC, in the group of adolescents with GAD. The healthy control adolescents showed opposite patterns of coupling between the amygdala and these regions. Our findings of negative connectivity between the amygdala and vmPFC within the AD group contribute to the growing body of evidence implicating disruption of the dynamic interplay within amygdala-PFC circuitry among individuals with anxiety disorders. Further, our results suggest this aberrant connectivity pattern can be observed at rest. Taken together, these findings suggest inefficient crosstalk between the amygdala and mPFC may lead to increased anxiety levels. Additional research will be needed in order to determine if this compromised connectivity is a defining feature of the underlying neurocircuitry of anxiety disorders.

In the current study, we observed altered connectivity between the amygdala and PCC, which is consistent with the growing body of literature linking disruption of this functional connection to mood and anxiety disorders [42,43,62]. Recent studies have implicated functional connections between the amygdala and posterior regions, such as the PCC and precuneus [19,29], in the implementation of emotional processing [62]. In addition, the PCC is a hub in the default mode network (DMN), a network that subserves processes such as mentalization and self-referential thinking [19,29], which may contribute to hypervigilance to interoceptive cues of anxiety. Indeed, prior studies have observed altered amygdala-PCC connectivity in pediatric GAD cohorts during emotional processing tasks [19,29] and at rest [48]. Taken together, these convergent findings suggest a tonic (task-independent) versus phasic (task-dependent) disruption in amygdala-PCC connectivity and future research will be needed to elucidate whether this is a defining neural underpinning of pediatric anxiety disorders. Recent work in depression has shown that treatment normalizes posterior cingulate-amygdala connectivity [52] and our findings suggest treatment targets for ADs and depression may overlap.

Amygdala-based connectivity correlated with PARS anxiety score across the entire sample, but this correlation was not significant within the AD group or HC group when considered independently, likely due to restriction of range. However, within the AD group, connectivity between the amygdala and PCC was positively correlated with age. Given this is the first documentation of this finding among youth and a cross-sectional study, we hesitate to over-interpret this finding. However, among HC youth, decreased connectivity between the amygdala and PCC has been observed across development [63]. The PCC is a key node in the DMN and default mode regions are known to functionally connect in a more integrated fashion across development [64], which may contribute to the current finding.

The current study is not without limitation. Although the sample size represents the largest to date, replication with a larger cohort of youth is necessary. However, the comorbidity profile of the current cohort may make our findings more generalizable, while noting that most patients (70%) had a GAD diagnosis. Participants in the current study met criteria for multiple ADs, similar to children presenting in clinical settings for treatment and to more recent clinical trials testing the efficacy of interventions in reducing overall anxiety. Moreover, although we observed one finding was significantly correlated with age, our sample size is underpowered for these analyses within the AD group. We captured a relatively wide age range in line with our desire to cast a wider net than previous studies. Data collection is ongoing, and a larger sample will allow for greater exploration of potential developmental effects. This larger sample may also allow for greater variability in anxiety levels within the AD group, making it more likely that variability in network functioning can be linked to severity of symptoms. We did not collect state anxiety symptoms at the time of the fMRI scan to relate to resting-state amygdala iFC. An additional limitation of the current data is that adolescent network functioning may differ from that of children and we look forward to future studies that can explore the nuances of healthy and disordered brain development. Clearly, the examination of the developmental trajectories of resting state networks among youth with and without ADs will be a groundbreaking work. Lastly, this is a cross-sectional observation and emotional face processing tasks (findings reported elsewhere) administered before the resting-state scan may have influenced connectivity in unexpected ways. Future work should address multiple resting state collection periods, acute influences of a preceding “emotional” task, as well as test order effects across tasks and rest, in order to determine the reliability of these networks.

## Conclusions

The current findings indicate that youth with ADs demonstrate altered intrinsic functional connectivity patterns, which has several implications. First, these results suggest dysfunction in the ALN, even in the absence of a specific anxiogenic task. Second, our results of aberrant connectivity are consistent with the adult literature. If network dysfunction is detectable early in the course of illness across youth with multiple ADs, then this may be a useful target for existing and novel treatments. This also opens the possibility that aberrant amygdala-frontal iFC is present early in the pathophysiology of pediatric anxiety and thus can serve as a potential biomarker of risk or illness development. Lastly, the examination of a brain marker across distinct primary AD diagnoses takes a step towards new dimensional construct of developmental psychopathology consistent with the Research Domain Criteria (RDoC) [65,66].

## Abbreviations

GAD: generalized anxiety disorder
AD: anxiety disorder
SP: social phobia
HC: healthy control
vmPFC: ventromedial prefrontal cortex
PCC: posterior cingulate cortex
ALN: anterior limbic network
SAD: separation anxiety disorder
CBT: cognitive behavioral therapy
mPFC: medial prefrontal cortex
ACC: anterior cingulate cortex
vlPFC: ventrolateral prefrontal cortex
dlPFC: dorsolateral prefrontal cortex
PARS: Pediatric Anxiety Rating Scale
rs-fMRI: resting-state functional magnetic resonance imaging
iFC: intrinsic functional connectivity
BOLD: blood-oxygen-level-dependent
AAL: automated anatomical labeling
DMN: default mode network
